# Editorial: The Evolution of Endothermy–From Patterns to Mechanisms

**DOI:** 10.3389/fphys.2018.00891

**Published:** 2018-07-12

**Authors:** Elias T. Polymeropoulos, Rebecca Oelkrug, Martin Jastroch

**Affiliations:** ^1^Institute for Marine and Antarctic Studies, University of Tasmania, Hobart, TAS, Australia; ^2^Department of Internal Medicine I, Group of Molecular Endocrinology, University of Lübeck, Lübeck, Germany; ^3^Institute for Diabetes and Obesity, Helmholtz Zentrum, Munich, Germany; ^4^The Arrhenius Laboratories F3, Department of Molecular Biosciences, The Wenner-Gren Institute, Stockholm University, Stockholm, Sweden

**Keywords:** endotherms, ectotherms, heterothermy, evolution, thermoregulation, comparative physiology

What makes endothermy one of the most fascinating traits in evolution? Metabolic heat production required a complex degree of coordination from the organism to the molecule to achieve a high, stable body temperature (T_b_), or homeothermic endothermy, which has convergently evolved as key trait of birds and mammals (Crompton et al., 1978). The resulting expansion of endotherms into thermal niches that were not accessible to ectotherms, whose T_b_ is dictated by ambient temperature (T_a_), proved to be the crucial evolutionary advantage. Yet, the evolutionary framework and events leading to endothermy are still unclear.

Generally, three streams of hypotheses have been proposed aiming to explain this extraordinary transition from ecto- to endothermy during vertebrate evolution. The first school of thought promotes that the thermoregulatory advantages of high T_b_s *per se*, resulting in higher metabolic rates (MR), suffice to explain the evolutionary advantage (Heinrich, 1977; Crompton et al., 1978). Others have put forward that the selection for higher MRs is a consequence of increased exercise, consequently raising T_b_ (Bennett and Ruben, 1979; Hayes and Garland, 1995). More recently, the parental care hypothesis has gained more traction (Farmer, 2000; Koteja, 2000), which stipulates an increase in T_b_ being beneficial for growth while reducing mortality, thus increasing species' fitness.

While this research topic aims at furthering and challenging these concepts through different methodological and theoretical approaches, it also provides novel experimental insights into physiological mechanisms on various levels of organization that may have underlay the transition to endothermy. The current issue also focuses on mechanisms that have enabled the maintenance of high, as well as fluctuations in T_b_ or MR in species that display local heterothermy or are facultative, homeothermic endotherms, thus filling missing links in our knowledge on why, how and when endothermy arose.

How is endothermy measured? Technically, measuring T_b_ or MR *per se*, despite being a common procedure, is not always a straight forward task. Detecting regional heterothermy and monitoring transitory states (e.g., hibernation) can be challenging, depending on the animal's body size, anatomy, and geographical distribution under free ranging conditions. However, characterizing metabolic phenotypes through thermometry is crucial to understand the evolution of different thermoregulatory strategies as well as linking genetic alterations to energy metabolism. The most commonly studied endotherms are small rodents and numerous thermometric techniques have been applied for metabolic phenotyping, whose accuracy and pitfalls are summarized by Meyer et al.

How did endothermy/ heterothermy impact mammalian evolution and survival? Previous hypotheses ascribe the survival of mammals during the mass extinction event at the K-Pg boundary to their heterothermic capacity (Lovegrove, 2016). Geiser et al. propose that this was achieved thanks to the ability of mammals to enter periods of reduced T_b_ and MR (torpor) induced by the wildfires of the asteroid impact and enabled some species to survive periods without food. In addition, such heterothermic states are considered beneficial during the transition to endothermy.

Tracing back the heterothermic ancestor giving rise to strict homeothermy has been a matter of a vivid debate. Not surprisingly, the holy grail in this quest has been the identification of heterothermic primates, whose presence appears restricted to the order of Strepsirrhini (Lovegrove, 2012). So far, no heterothermic relatives in the sister clades of Haplorrhini have been identified, including tarsiers (Welman et al.), the closest extant relative to Strepsirrhini.

Fluctuations in T_b_ is very common among facultative endotherms, who display regional heterothermy, torpor or hibernation (Angilletta et al., 2010; Ruf and Geiser, 2015). The question thus arises whether reaction rates of biochemical processes in endotherms are plastic and thus adaptable to T_a_ fluctuations. Seebacher and Little consider reaction rates of endotherms as thermally plastic, a trait that may be conserved from ectothermic ancestors. Amongst others, this notion is based on cellular pathways responding to thermal cues in tissues, like the transient potential receptor ion channels (TRPV and TRPM) and the AMP-activated protein kinase (AMPK) that affect energy expenditure.

The lesser hedgehog tenrec (*Echinops telfairi*) displays highly pronounced, reptile like thermoregulatory flexibility, which may be indicative of its phylogeny, representing a transitory state between ecto- and endotherms called basoendotherms. Polymeropoulos et al. show that this thermoregulatory flexibility is supported by the maintenance of the efficiency of mitochondrial ATP production (coupling efficiency). In particular the reduction of the basal mitochondrial proton leak compensates the reduction in substrate oxidation capacity at low temperatures.

How do mammals adapt heat production? Brown adipose tissue (BAT) mediated, nonshivering thermogenesis (NST) has long been recognized as a key thermogenic mechanism of small placental mammals and neonates that has proven as an evolutionary and thermoregulatory advantage in cold climates (Cannon and Nedergaard, 2004). The high concentration of mitochondria and expression of uncoupling protein 1 (UCP1) in BAT is crucial to the generation of heat. Therefore, understanding the molecular control of UCP1 expression and its evolution by identifying transcriptional regulatory elements across the mammalian phylogeny beyond murid rodents is essential to understand how UCP1 and BAT have been integrated allowing cellular heat production. The recent, most comprehensive analysis of UCP1 regulatory elements in mammals so far (Gaudry and Campbell), identifies various regulatory genomic elements and regions which appear less crucial than suggested previously, while highlighting others like the proximal TATA box and the distal enhancer region which are universally in control of intact UCP1 orthologs. This ground work allows targeted, comparative studies into UCP1 regulation that may shed new light into the evolution of adaptive thermogenesis in the vertebrate lineage (Fromme).

While numerous studies consider the evolution of BAT as a critical mechanism during the evolution toward endothermy, a separate BAT-independent mechanism of NST in skeletal muscle has been proposed (Bal et al., 2012). Here heat dissipation relies on Ca^2+^-slippage by a sarcoplasmatic reticulum Ca^2+^-ATPase (SERCA) that is controlled by sarcolipin (SLN). Nowack et al. summarize support to the hypothesis that muscle based NST is the earliest form of NST giving rise to endothermy, rather than BAT-mediated NST, which in addition to muscle-based NST, supported heterothermic endotherms during their early evolution. In stark contrast, Campbell and Dicke argue that evidence for an adaptive, thermogenic role (especially in larger bodied mammals) of the interaction between SLN and SERCA, regulating Ca^2+^-slippage, is inconclusive so far and that more comparative analyses on SLN expression across multiple taxa are required to uncover its physiological role during mammalian evolution.

The large amounts of energy expended by endotherms in order to maintain high T_b_s, especially in cold climates, not only appears energetically wasteful (Koteja, 2004) but also demands an efficient machinery minimizing the production of potentially harmful byproducts. Tissues of high aerobic capacity may produce reactive oxygen species (ROS) in excess when e.g., muscular work is performed in order to maintain a higher T_b_ such as in endothermic fish. These ROS may cause macromolecular damage and are shown to increase with increasing T_a_ (Banh et al., 2016). However, ROS production in red muscle of endothermic tuna is similar to ectothermic fish when measured at the physiological temperature, indicating the lack of compensatory mechanisms for increased ROS production, that may have coevolved with endothermy (Wiens et al.).

The advent of modern molecular tools allowing the formulation of comparative and functional genomic and quantitative genetic models (Nespolo et al., 2011) adds support for the parental care hypothesis (Bacigalupe et al.). Here a quantitative genetic model of maternal effects suggests that an increase in basal metabolic rate (BMR) and T_b_ (based on increased size/ activity of visceral organs to maintain elevated assimilation rates) may have been the result of their natural selection since there is a positive covariance between growth rate and the daily energy expenditure.

In support of the aerobic capacity model, previous work by Sadowska et al. (2015) in bank voles has shown that selection for high aerobic metabolism leads to an increase in BMR and thermogenic capacity. Following this work, Stawski et al. now demonstrate that this adaptation also affects the thermoregulatory curve through an increase in RMR as well as T_b_ but also increases maximum thermogenesis yielding the selected lines more cold tolerant.

Endothermic T_b_s reach their peak within birds, exceeding those of mammals by up to 2.5°C (Prinzinger et al., 1991). These extraordinarily high T_b_s pose a significant problem for migrating birds because of the large amount of work performed and concomitant muscular heat produced during flight, increasing the risk of hyperthermia. Consequently large eider ducks (*Somateria mollissima*) adapt their migration strategy to prevent hyperthermia by decreasing in flight duration (Guillemette et al., 2016) and prolonged periods of cooling during breaks, resulting in a behavioral stop-and-go, thermoregulatory strategy that poses a significant migratory time cost (Guillemette et al.).

Neural control of mammalian thermoregulation has been well defined, including pathways eliciting shivering as well as non-shivering thermogenic responses (Morrison and Nakamura, 2011). Strikingly however, thermoregulation appears to be impaired during mammalian REM sleep (Parmeggiani, 2003) a phenomenon which is less well understood. Cerri et al. conclude that REM sleep is a transient heterothermic state that coevolved with endothermy, which benefits regeneration of brain activity rather than energy conservation.

The outstanding contributions to this research topic will refine our understanding of when, how and why the evolution of endothermy emerged. The diversity of approaches, fusing technological advances, ecological, physiological and molecular insights, complement the picture on the evolution of endothermy–that still remains one of the patchiest mysteries of nature. The use of modern molecular and comparative techniques will allow an acceleration in the understanding of the evolution of endothermy that will, however, likely raise just as many questions as it will answer in the future.

## Author contributions

EP, RO, and MJ contributed to manuscript writing, provided important interpretations, critically revised the work, and provided final approval of the opinion content.

### Conflict of interest statement

The authors declare that the research was conducted in the absence of any commercial or financial relationships that could be construed as a potential conflict of interest.
